# Existence of a Unique Solution to a Fractional Partial Differential Equation and Its Continuous Dependence on Parameters

**DOI:** 10.3390/e23070851

**Published:** 2021-07-01

**Authors:** Robert Stegliński

**Affiliations:** Institute of Mathematics, Lodz University of Technology, Wólczańska 215, 90-924 Lodz, Poland; robert.steglinski@p.lodz.pl

**Keywords:** monotone operator, unique solution, Lyapunov-type inequalities, dependence on parameters

## Abstract

In the present paper we give conditions under which there exists a unique weak solution for a nonlocal equation driven by the integrodifferential operator of fractional Laplacian type. We argue for the optimality of some assumptions. Some Lyapunov-type inequalities are given. We also study the continuous dependence of the solution on parameters. In proofs we use monotonicity and variational methods.

## 1. Introduction

In this paper, we study the existence of a unique solution to a nonlocal equation

(1) $$\left\{\begin{array}{cc} -L_{K}u=f\left(x,u\right) & \mathrm{in}\;\Omega \\ u=0 & \mathrm{in}\;R^{N}\backslash \Omega , \end{array}\right.$$

where $\Omega \subset \;R^{N},\;N\geq 2,$ is a bounded domain with Lipschitz boundary ∂Ω and f:Ω×R→R is a function verifying some suitable conditions. The integrodifferential operator of fractional Laplacian type $L_{K}$ is defined pointwise by

$$L_{K}u\left(x\right)=\int_{R^{N}}\left[u\left(x+y\right)+u\left(x-y\right)-2u\left(x\right)\right]K\left(y\right)dy,\;\;\;\;\;x\in R^{N},$$

where the kernel $K:R^{N}\backslash \left\{0\right\}\to \left(0,+\infty \right)$ satisfies the following:

(*K*1) $\gamma K\in L^{1}\left(\Omega \right),$ where $\gamma \left(x\right)=\min\left\{{\left|x\right|}^{2},1\right\}$;
(*K*2) there exist λ>0 and s∈(0,1) such that $K\left(x\right)\geq \lambda {\left|x\right|}^{-(N+2s)}$ for all $x\in R^{N}\backslash \left\{0\right\}.$

A typical example for *K* is given by $K\left(x\right)={\left|x\right|}^{-(N+2s)}$. In this case $L_{K}$ is the so-called fractional Laplacian operator ${\left(-\Delta \right)}^{s},$ which, up to multiplying by a suitable constant, has the following property:

$$\lim_{s\to 1^{-}}{\left(-\Delta \right)}^{s}u=-\Delta u.$$

For this fact and more details on the fractional Laplacian, see [1]. Let us also mention some important works concerning problems with the integrodifferential operator of fractional Laplacian type (i.e., [2,3,4,5,6]).

Recently, a great deal of attention has been focused on the study of non-local elliptical operators, both for pure mathematical research and in view of concrete applications, since these operators arise quite naturally in many different contexts, such as the thin obstacle problem [7], optimization [8], finance [9], phase transitions [10], stratified materials [11], anomalous diffusion [12], crystal dislocation [13], semipermeable membranes and flame propagation [14], conservation laws [15], ultra-relativistic limits of quantum mechanics [16], quasi-geostrophic flows [17], multiple scattering [18], minimal surfaces [19], materials science [20], and water waves [21]. These applications force us to follow Courant and Hilbert (see ([22], p. 227)): “*A mathematical problem which is to correspond to physical reality should satisfy the following basic requirements: (1) The solution must exist. (2) The solution should be uniquely determined. (3) The solution should depend continuously on the data (requirement of stability).*” In this work we will meet all the above requirements. First we give stipulations on the source *f* under which the problem has exactly one weak solution. In the proof we use the Minty–Browder theorem. We show that some monotonicity conditions on the source *f* are optimal. To the best of our knowledge, there are no such optimal results for nonlocal problems. Our approach is based on the work [23]. As a byproduct we obtain some Lyapunov-type inequalities. Next, we show continuous dependence of the solution on parameters. In proofs we use monotonicity and variational methods (see [24,25,26]).

## 2. Preliminaries

To define our solution space $X_{0}$, we define the fractional Sobolev space

$$X=\{u:R^{N}\to R\;s.t.\;u\;\mathrm{is}\;\mathrm{Lebesgue}\;\mathrm{measurable},\;u{|}_{\Omega }\in L^{2}\left(\Omega \right)$$

$$\;\mathrm{and}\;\int_{Q}{\left|u(x)-u(y)\right|}^{2}K\left(x-y\right)dxdy<\infty \},$$

where $Q=R^{2N}\backslash \left(\Omega^{c}\times \Omega^{c}\right),\Omega^{c}=R^{N}\backslash \Omega ,$ and we restrict it to the subspace

$$X_{0}=\left\{u\in X:u\left(x\right)=0\;\;\mathrm{for}\;a.a.\;\;x\in R^{N}\backslash \Omega \right\},$$

which is a separable Hilbert space under the norm

$$∥u∥={\left(\int_{Q}{\left|u(x)-u(y)\right|}^{2}K\left(x-y\right)dxdy\right)}^{1/2}={\left(\int_{R^{2N}}{\left|u(x)-u(y)\right|}^{2}K\left(x-y\right)dxdy\right)}^{1/2}$$

induced by the inner product

$$\left(u,v\right)=\int_{R^{2N}}\left(u\left(x\right)-u\left(y\right)\right)\left(v\left(x\right)-v\left(y\right)\right)K\left(x-y\right)dxdy.$$

We denote by $X^{*}$ the topological dual of $X_{0}.$ The critical exponent is defined as $2_{s}^{*}=\frac{2N}{N-2s}$, and the embedding $i:X_{0}\to L^{p}\left(\Omega \right)$ is continuous for all $p\in [1,2_{s}^{*}]$ and compact for all $p\in [1,2_{s}^{*}).$ For $p\in [1,2_{s}^{*}]$ define a constant

(2) $$c_{p}=\underset{X_{0}\backslash \left\{0\right\}}{\inf}\frac{∥u∥}{{∥u_{|\Omega }∥}_{p}}.$$

Here ${∥\cdot ∥}_{p}$ denotes the standard norm in $L^{p}\left(\Omega \right).$

An element $u\in X_{0}$ is called a weak solution of problem (1) if $f\left(\cdot ,u\left(\cdot \right)\right)\in L^{p^{\prime }}\left(\Omega \right),$ where $p^{\prime }=\frac{p}{p-1}$ for some $p\in (1,2_{s}^{*}]$ and we have

$$\int_{R^{2N}}\left(u\left(x\right)-u\left(y\right)\right)\left(v\left(x\right)-v\left(y\right)\right)K\left(x-y\right)dxdy=\int_{\Omega }f\left(x,u\right)v\;dx$$

for all $v\in X_{0}$.

## 3. Uniqueness

Now we present conditions under which the problem (1) has exactly one weak solution. Recall that f:Ω×R→R is a Carathéodory function if f(x,·) is continuous for a.a. x∈Ω and f(·,t) is measurable for all t∈R. For p∈[1,∞], let $p^{\prime }\in \left[1,\infty \right]$ be such that $\frac{1}{p}+\frac{1}{p^{\prime }}=1.$ Note that if $\frac{N}{2s}\leq p\leq \infty ,$ then $2\leq 2p^{\prime }\leq 2_{s}^{*}.$ We impose the following assumptions on function f:Ω×R→R:

(F) There exist $\eta \in L^{{\left(2_{s}^{*}\right)}^{\prime }}\left(\Omega \right)$ and α>0 such that
  $$\left|f(x,t)\right|\leq \eta \left(x\right)+\alpha {\left|t\right|}^{2_{s}^{*}-1}$$
  for a.a. x∈Ω and all t∈R;
(A) There exists a measurable a:Ω→R such that
  (3) $$\left(f\left(x,t_{1}\right)-f\left(x,t_{2}\right)\right)\left(t_{1}-t_{2}\right)\leq a\left(x\right){\left(t_{1}-t_{2}\right)}^{2}$$
  for a.a. x∈Ω and all $t_{1},t_{2}\in R$.

**Remark** **1.** *If the function f:Ω×R→R is a $C^{1}$-Carathéodory function, that is, f(x,·) is of class $C^{1}$ for a.a. x∈Ω and $f\left(\cdot ,t\right),f_{t}^{\prime }\left(\cdot ,t\right)$ are measurable for all t∈R, then the inequality (3) is equivalent to*

$$f_{t}^{\prime }\left(x,t\right)\leq a\left(x\right)$$

*for a.a. x∈Ω and all t∈R.*

**Theorem** **1.** *Let f:Ω×R→R be a Carathéodory function satistying (F) and (A) with $a\in L^{p}\left(\Omega \right),\frac{N}{2s}\leq p\leq \infty ,$ such that*

(4) $${∥a_{+}∥}_{p}<c_{2p^{\prime }}^{2},$$

*where the constant $c_{2p^{\prime }}$ is defined in (2) and $a_{+}=\max\left\{a,0\right\}$. Then the problem (1) has a unique weak solution $u_{0}$ in $X_{0}$ and its norm satisfies the following estimation from above*

(5) $$∥u_{0}∥\leq \frac{c_{2_{s}^{*}}{∥\eta ∥}_{{\left(2_{s}^{*}\right)}^{\prime }}}{1-c_{2p^{\prime }}^{-2}{∥a_{+}∥}_{p}}.$$

*Moreover, if $\frac{N}{2s}<p\leq \infty ,$ then the constant in (4) is optimal in the sense that there exists $a\in L^{p}\left(\Omega \right)$ with ${∥a_{+}∥}_{p}=c_{2p^{\prime }}^{2}$ such that the problem (1) with f(x,u)=a(x)u has at least two weak solutions.*

**Proof.**  Clearly, the weak solvability of the problem (1) is equivalent to the solvability of equation T(u)=0 in $X_{0},$ where $T:X_{0}\to X^{*}$ is defined by

$$\langle T(u),v\rangle =\int_{R^{2N}}\left(u\left(x\right)-u\left(y\right)\right)\left(v\left(x\right)-v\left(y\right)\right)K\left(x-y\right)dxdy-\int_{\Omega }f\left(x,u\right)v\;dx$$

for all $u,v\in X_{0}.$ Here, $\langle \cdot ,\cdot \rangle$ denotes the duality brackets for the pair $\left(X^{*},X_{0}\right)$. Note that the second part in the definition of *T* can be represented in the form ${\langle N_{f}\left(iu\right),iu\rangle }_{L^{{\left(2_{s}^{*}\right)}^{\prime }}\left(\Omega \right),L^{2_{s}^{*}}\left(\Omega \right)},$ where $i:X_{0}\to L^{2_{s}^{*}}\left(\Omega \right)$ is continuous embedding, $N_{f}:L^{2_{s}^{*}}\left(\Omega \right)\to L^{{\left(2_{s}^{*}\right)}^{\prime }}\left(\Omega \right),$ $N_{f}\left(u\right)=f\left(\cdot ,u\right)$ is the Nemytskii map, which is continuous by assumption (F) and ([27], Proposition 2.76), and ${\langle \cdot ,\cdot \rangle }_{L^{{\left(2_{s}^{*}\right)}^{\prime }}\left(\Omega \right),L^{2_{s}^{*}}\left(\Omega \right)}$ denotes the duality brackets for the pair $(L^{{\left(2_{s}^{*}\right)}^{\prime }}\left(\Omega \right),L^{2_{s}^{*}}\left(\Omega \right)).$ Hence, the operator *T* is well defined and continuous.

Now, assumption (A) with the Hölder inequality gives

(6) $$\begin{array}{ccc} \langle T(u)-T(v),u-v\rangle & = & {∥u-v∥}^{2}-\int_{\Omega }(f\left(x,u\right)-f\left(x,v\right)\left(u-v\right)dx\\ & \geq & {∥u-v∥}^{2}-\int_{\Omega }a_{+}\left(x\right){\left(u-v\right)}^{2}dx\\ & \geq & {∥u-v∥}^{2}-{∥a_{+}∥}_{p}{∥u-v∥}_{2p^{\prime }}^{2}\\ & \geq & \left(1-c_{2p^{\prime }}^{-2}{∥a_{+}∥}_{p}\right){∥u-v∥}^{2} \end{array}$$

for all u,v∈$X_{0}.$ This and (4) show that *T* is strongly monotone, and so *T* is strictly monotone and coercive (see ([24], p. 501). Then the equation T(u)=0 has a unique solution $u_{0}\in X_{0}$ by the Minty–Browder theorem (see ([24], Theorem 26A)). Moreover, putting in (6) $u=u_{0}$ and v=0, since $T(u_{0})=0,$ we obtain

$$\begin{array}{ccc} \left(1-c_{2p^{\prime }}^{-2}{∥a_{+}∥}_{p}\right){∥u_{0}∥}^{2} & \leq & \langle -T\left(0\right),u_{0}\rangle =\int_{\Omega }f\left(x,0\right)u_{0}\;dx\\ & \leq & {∥\eta ∥}_{{\left(2_{s}^{*}\right)}^{\prime }}{∥u_{0}∥}_{2_{s}^{*}}\leq c_{2_{s}^{*}}{∥\eta ∥}_{{\left(2_{s}^{*}\right)}^{\prime }}∥u_{0}∥, \end{array}$$

which gives (5).

Now, let $\frac{N}{2s}<p\leq \infty .$ Then $2\leq 2p^{\prime }<2_{s}^{*}.$ Define $J_{p}:X_{0}\backslash \left\{0\right\}\to R$ by

$$J_{p}\left(u\right)=\frac{{∥u∥}^{2}}{{∥u∥}_{2p^{\prime }}^{2}}=\frac{\int_{R^{2N}}{\left(u\left(x\right)-u\left(y\right)\right)}^{2}K\left(x-y\right)dxdy}{{\left(\int_{\Omega }{\left|u\right|}^{2p^{\prime }}\right)}^{\frac{1}{p^{\prime }}}}.$$

Then $\inf_{X_{0}\backslash \left\{0\right\}}J_{p}=c_{2p^{\prime }}^{2}.$ Let $\left\{u_{n}\right\}\subset X_{0}\backslash \left\{0\right\}$ be a minimizing sequence. Since $J_{p}$ is homogeneous of degree 0, we can assume without loss of generality that

$${∥u_{n}∥}_{2p^{\prime }}=1\;\;\;\mathrm{and}\;\;\;∥u_{n}∥\to c_{2p^{\prime }}$$

as n→∞. Hence, $\left\{u_{n}\right\}$ is bounded in $\;X_{0}.$ Thus we can choose a subsequence (denoted again by the same symbol $\left\{u_{n}\right\}$) and a function $u_{0}\in X_{0}$ such that $u_{n}⇀u_{0}$ in $X_{0}$ and $u_{n}\to u_{0}$ strongly in $L^{2p^{\prime }}\left(\Omega \right).$ The strong convergence in $L^{2p^{\prime }}\left(\Omega \right)$ gives us ${∥u_{0}∥}_{2p^{\prime }}=1.$ The weak convergence in $X_{0}$ implies

$$J_{p}\left(u_{0}\right)\leq \lim\inf J_{p}\left(u_{n}\right)=c_{2p^{\prime }}^{2}.$$

Then $u_{0}\in$$X_{0},u_{0}\neq 0,$ is a minimizer of $J_{p}.$

Clearly, $u_{0}$ also minimizes the differentiable functional $I_{p}$$:X_{0}\to R$ defined by

$$I_{p}\left(u\right)=\int_{R^{2N}}{\left(u\left(x\right)-u\left(y\right)\right)}^{2}K\left(x-y\right)dxdy-c_{2p^{\prime }}^{2}{\left(\int_{\Omega }{\left|u\right|}^{2p^{\prime }}\right)}^{\frac{1}{p^{\prime }}}.$$

Thus $I_{p}^{\prime }\left(u_{0}\right)\left(v\right)=0$ for all v∈$X_{0},$ that is,

$$\int_{R^{2N}}\left(u_{0}\left(x\right)-u_{0}\left(y\right)\right)\left(v\left(x\right)-v\left(y\right)\right)K\left(x-y\right)dxdy-c_{2p^{\prime }}^{2}{\left(\int_{\Omega }{\left|u_{0}\right|}^{2p^{\prime }}\right)}^{\frac{1}{p^{\prime }}-1}\int_{\Omega }{\left|u_{0}\right|}^{2p^{\prime }-2}u_{0}v=0$$

for all v∈$X_{0}.$ If we put a(x)=$c_{2p^{\prime }}^{2}{\left(\int_{\Omega }{\left|u_{0}\right|}^{2p^{\prime }}\right)}^{\frac{1}{p^{\prime }}-1}{\left|u_{0}\left(x\right)\right|}^{2p^{\prime }-2}$ for all x∈Ω, then $u_{0}$ is a nontrivial weak solution for problem (1) with f(x,u)=a(x)u for all x∈Ω and u∈R. Moreover, ${∥a_{+}∥}_{p}=\;c_{2p^{\prime }}^{2}.$ Since now the problem (1) is linear, the zero function is a second solution. This proves the theorem. □

**Remark** **2.** Let us note that if we want to obtain a unique solution by using monotonicy methods with the Minty–Browder theorem, then the operator related to our problem must be strictly monotone, coercive, and continuous. For the operator to be well defined and continuous, we need the assumption (F) that is optimal. For strict monotonicity and coercivity, we need the source f to increase linearly at most, where the coefficient may depend on x. If we treat this coefficient as a function in $L^{p},$ we have obtained the optimal bound on its p-norm with $\frac{N}{2s}<p\leq \infty$. Note also that, if f(x,t)=a(x)t with $a\in L^{\frac{N}{2s}}\left(\Omega \right),$ then f satisfies (F) with $\alpha =\left(N-2s\right){\left(N+2s\right)}^{\frac{N+2s}{2s-N}}{\left(4s\right)}^{\frac{2N}{2s-N}}$ and $\eta =a^{\frac{N+2s}{4s}},$ which belongs to $L^{{\left(2_{s}^{*}\right)}^{\prime }}\left(\Omega \right).$

Theorem 1 allows us to give Lyapunov-type inequalities for a linear problem

(7) $$\left\{\begin{array}{cc} -L_{K}u=a\left(x\right)u & \mathrm{in}\;\Omega \\ u=0 & \mathrm{in}\;R^{N}\backslash \Omega , \end{array}\right.$$

that is, a necessary condition on *a* for the existence of a non-trivial solution *u* to the problem (7).

**Corollary** **1.** *Let 0<s<1 and $\frac{N}{2s}\leq p\leq \infty .$ If problem (7) has a non-trivial solution, then*

$${∥a_{+}∥}_{p}\geq c_{2p^{\prime }}^{2}$$

*and the inequality is optimal if $p>\frac{N}{2s}$. Moreover, if $p>\frac{N}{2s}$ and M is any positive number, then there exists $a\in L^{p}\left(\Omega \right)$ such that ${∥a_{+}∥}_{p}>M$ and problem (7) has only a trivial solution.*

**Proof.**  By the first part of Theorem 1, if *a* in problem (7) satisfies ${∥a_{+}∥}_{p}<c_{2p^{\prime }}^{2},$ then there is exactly one solution to (7), so it is a trivial one. The optimality follows from the second part of Theorem 1. Now fix $p>\frac{N}{2s}$ and take any M>0. Next, choose $\frac{N}{2s}<q<p.$ From properties of Lebesgue spaces we can find a function $a\in L^{p}\left(\Omega \right)$ such that ${∥a_{+}∥}_{q}<c_{2q^{\prime }}^{2}$ and ${∥a_{+}∥}_{p}>M.$ With such a function, (7) has only trivial solution, by Theorem 1. □

In the notation of [28], we can present this result as follows. For 0<s<1 put

$$\begin{array}{ccc} \Lambda_{s} & = & \{a\in L^{\frac{N}{2s}}\left(\Omega \right):(7)\;\mathrm{has}\;a\;\mathrm{nontrivial}\;\mathrm{solution}\},\\ V_{s} & = & \left\{a\in L^{\frac{N}{2s}}\left(\Omega \right):(7)\;\mathrm{has}\;\mathrm{no}\;\mathrm{nontrivial}\;\mathrm{solutions}\right\} \end{array}$$

and for $\frac{N}{2s}\leq p\leq \infty$ define the value

$$\begin{array}{ccc} \beta_{p,s} & = & \underset{a\in \Lambda_{s}\cap L^{p}\left(\Omega \right)}{\inf}{∥a_{+}∥}_{p}\\ \gamma_{p,s} & = & \underset{a\in V_{s}\cap L^{p}\left(\Omega \right)}{\sup}{∥a_{+}∥}_{p}. \end{array}$$

Now Corollary 1 takes the following form.

**Corollary** **2.** *Let 0<s<1.*

*1*. $\beta_{\frac{N}{2s},s}\geq c_{2_{s}^{*}}^{2}$;
*2*. *$\beta_{p,s}=c_{2p^{\prime }}^{2}$ for $\frac{N}{2s}<p\leq \infty$;*
*3*. *$\gamma_{p,s}=+\infty$ for $\frac{N}{2s}<p\leq \infty .$*

Now, applying ([29], Theorem 3.3) we have an estimate from below for $\beta_{p,s}$ with 0<s<1 and $\frac{N}{2s}<p<\infty$:

$$\beta_{p,s}\geq \frac{C}{r_{\Omega }^{2s-\frac{N}{p}}},$$

where *C* depends only on s,p, and N, and $r_{\Omega }$ is the inner radius of Ω, that is, $r_{\Omega }=\max_{x\in \Omega }\inf_{y\in \partial \Omega }\left|x-y\right|.$ This and Corollary 2 give us an estimate from below for $c_{p}$:

$$c_{p}\geq \frac{C}{r_{\Omega }^{s-\frac{N(p-2)}{2p}}},$$

where $2\leq p<2_{s}^{*}.$

If we consider problem (7) driven by the Laplacian operator, $\Omega \subset R^{N}$ is a ball and N≥3, then $\beta_{p,1}=0$ for $1\leq p<\frac{N}{2},$ by ([30], Theorem 2.5). A natural question then arises.

**Open problem** *Is it true that* $\beta_{p,s}=0$ *for* 0<s<1 *and* $1\leq p<\frac{N}{2s}$ *? What is the value of* $\gamma_{p,s}$ *for* 0<s<1 *and* $1\leq p\leq \frac{N}{2s}$ *?*

**Remark** **3.** *Similarly to two-point boundary value problems (see [31]), we say that problem (1) across many resonant points if $f\in C^{1}\left(\Omega \times R\right)$ and the range of $f_{t}^{\prime }$, the derivative of f with respect to the second argument, contains many eigenvalues of the differential operator of the problem. By Theorem 1, we can find problems (1) with unique solution, across as many resonant points as we wish. Indeed, for any finite set of eigenvalues we can find $a\in C^{1}\left(\Omega \right)$ containig in its range this set of eigenvalues and such that ${∥a∥}_{p}<c_{2p^{\prime }}^{2}$ for some $p>\frac{N}{2s}$ and so problem (7) with this a has a unique weak solution.*

## 4. Continuous Dependence on Parameters

In this section we consider a problem with parameters. For a fixed set Σ⊂R and q∈[1,∞) put

$$L_{\Sigma }^{q}=\left\{w\in L^{q}\left(\Omega \right):w\left(x\right)\in \Sigma \;\;\mathrm{for}\;a.a.\;x\in \Omega \right\}.$$

The set $L_{\Sigma }^{q}$ will be termed the set of admissible parameters. Consider the following problem, which is subject to a parameter $w\in L_{\Sigma }^{q}$

(8) $$\left\{\begin{array}{cc} -L_{K}u=f\left(x,u,w\right) & \mathrm{in}\;\Omega \\ u=0 & \mathrm{in}\;R^{N}\backslash \Omega . \end{array}\right.$$

where f:Ω×R×Σ→R is a Carathéodory function, that is, (x,u,w)↦f(x,u,w) is continuous in (u,w) for a.a. x∈Ω and measurable in *x* for each (u,w)∈R×Σ.

An element $u\in X_{0}$ is a weak solution of problem (8) if

$$\int_{R^{2N}}\left(u\left(x\right)-u\left(y\right)\right)\left(v\left(x\right)-v\left(y\right)\right)K\left(x-y\right)dxdy=\int_{\Omega }f\left(x,u\left(x\right),w\left(x\right)\right)v\left(x\right)dx$$

for all $v\in X_{0}$.

We need to modify assumptions (A) and (F) in the following way:

(F*_w_*) There exist $r\in (1,2_{s}^{*}),$ $\eta \in L^{r^{\prime }}\left(\Omega \right)$ and $\alpha_{1},\alpha_{2}>0$ such that
  $$\left|f(x,t,w)\right|\leq \eta \left(x\right)+\alpha_{1}{\left|t\right|}^{r-1}+\alpha_{2}{\left|w\right|}^{q/r^{\prime }}$$
  for a.a. x∈Ω and all (t,w)∈R×Σ.
(A*^w^*) There exist a measurable a:Ω→R such that
  $$\left(f\left(x,t_{1},w\right)-f\left(x,t_{2},w\right)\right)\left(t_{1}-t_{2}\right)\leq a\left(x\right){\left(t_{1}-t_{2}\right)}^{2}$$
  for a.a. x∈Ω and all $t_{1},t_{2}\in R,w\in \Sigma$.

Note that if *f* satisfies assumptions ($F_{w}$) and ($A_{w}$), then for a fixed parameter $w\in L_{\Sigma }^{q},$q∈[1,∞), the function *f* satisfies (F) and (A), and so the problem (8) has a unique weak solution $u\in X_{0}$, by Theorem 1. In this case we say that the solution *u* corresponds to the parameter w. So, assuming ($A_{w}$) and ($F_{w}$), we define a single-valued solution operator $S_{f}:L_{\Sigma }^{q}\to X_{0},$ which assigns to any parameter $w\in L_{\Sigma }^{q}$ the unique weak solution $u\in X_{0}$ of problem (8). If $L_{\Sigma }^{q}$ is considered to be endowed with the relative topology induced from the topology on $L^{q}\left(\Omega \right),$ we can formulate the following theorem on continuous dependence on parameters.

**Theorem** **2.** *Assuming ($F_{w}$) and ($A_{w}$) with $a\in L^{p}\left(\Omega \right),\frac{N}{2s}\leq p\leq \infty ,$ such that ${∥a_{+}∥}_{p}<c_{2p^{\prime }}^{2}$, the solution operator $S_{f}:L_{\Sigma }^{q}\to X_{0}$ is continuous.*

**Proof.**  Let $\left\{w_{k}\right\}\subset L_{\Sigma }^{q}$ be a sequence of admissible parameters convergent in $L^{q}\left(\Omega \right)$ to $w_{0}\in L_{\Sigma }^{q}$ and let $u_{k}\in X_{0}$ be the unique solution to problem (8) corresponding to the parameter $w_{k},$ that is, $u_{k}=S_{f}\left(w_{k}\right),$ for each k∈N∪{0}. Choose any subsequence ${\left\{u_{k_{n}}\right\}}_{n\in N}$ of ${\left\{u_{k}\right\}}_{k\in N}$. To the end, it is enough to show that ${\left\{u_{k_{n}}\right\}}_{n\in N}$ has a convergent subsequence.

First, we show that ${\left\{u_{k}\right\}}_{k\in N}$ is bounded in $X_{0}$. For any k∈N∪{0} define $T_{k}:X_{0}\to X^{*}$ by

$$\langle T_{k}\left(u\right),v\rangle =\left(u,v\right)-\int_{\Omega }f\left(x,u,w_{k}\right)v\;dx\;\;\;\;\;\mathrm{for}\;\mathrm{all}\;\;u,v\in X_{0},$$

that is, the operator defined in the proof of Theorem 1. We have $T_{k}\left(u_{k}\right)=0,$ since $u_{k}$ is the weak solution corresponding to parameter $w_{k}.$ Clearly, for each fixed parameter *w*, ($F_{w}$) implies (F), so we can use the proof of Theorem 1. So, using (6) with $u=u_{k}$ and v=0, we obtain

$$\begin{array}{ccc} \left(1-c_{2p^{\prime }}^{-2}{∥a_{+}∥}_{p}\right){∥u_{k}∥}^{2} & \leq & -\langle T_{k}\left(0\right),u_{k}\rangle =\int_{\Omega }f\left(x,0,w_{k}\right)u_{k}\;dx\\ & \leq & \int_{\Omega }\left(\eta \left|u_{k}\right|+{\left|w_{k}\right|}^{q/r^{\prime }}\left|u_{k}\right|\right)\;dx\\ & \leq & \left({∥\eta ∥}_{r^{\prime }}+{∥w_{k}∥}_{q}^{q/r^{\prime }}\right){∥u_{k}∥}_{r}\\ & \leq & c_{r}\left({∥\eta ∥}_{r^{\prime }}+{∥w_{k}∥}_{q}^{q/r^{\prime }}\right)∥u_{k}∥. \end{array}$$

The boundedness of ${\left\{{∥w_{k}∥}_{q}\right\}}_{k\in N}$ implies the boundedness of ${\left\{u_{k}\right\}}_{k\in N}$ in $X_{0}.$ Hence ${\left\{u_{k_{n}}\right\}}_{n\in N}$ has a subsequence, denoted again by ${\left\{u_{k_{n}}\right\}}_{n\in N},$ weakly convergent to some ${\tilde{u}}_{0}\in X_{0},$ that is,

(9) $$\left(u_{k_{n}},v\right)⟶\left({\tilde{u}}_{0},v\right)\;\;\mathrm{for}\;\mathrm{all}\;v\in X_{0}.$$

Since the embedding $X_{0}\to L^{r}\left(\Omega \right)$ is compact, we have—up to a subsequence—that

$$u_{k_{n}}⟶{\tilde{u}}_{0}\;\;\mathrm{in}\;L^{r}\left(\Omega \right)$$

and there exists $h_{1}\in L^{r}\left(\Omega \right)$ such that, almost everywhere on Ω, $\left|u_{k_{n}}\left(x\right)\right|,\left|{\tilde{u}}_{0}\left(x\right)\right|\leq h_{1}\left(x\right)$ and $u_{k_{n}}\left(x\right)⟶{\tilde{u}}_{0}\left(x\right)$ (see ([32], Lemma A.1)). Moreover, since $w_{k_{n}}\to w_{0}$ in $L^{q}\left(\Omega \right),$ we have, up to a subsequence, $w_{k_{n}}\left(x\right)\to w_{0}\left(x\right)$ and $\left|w_{k_{n}}\left(x\right)\right|,\left|w_{0}\left(x\right)\right|\leq h_{2}\left(x\right)$ for a.a. x∈Ω and some $h_{2}\in L^{q}\left(\Omega \right).$ Considering all this and our assumptions, we obtain

$$f\left(\cdot ,u_{k_{n}}\left(\cdot \right),w_{k_{n}}\left(\cdot \right)\right)u_{k_{n}}\left(\cdot \right)-f\left(\cdot ,{\tilde{u}}_{0}\left(\cdot \right),w_{0}\left(\cdot \right)\right){\tilde{u}}_{0}\left(\cdot \right)\to 0\;\;\mathrm{almost}\;\mathrm{everywhere}\;\mathrm{on}\;\Omega$$

and

$$\left|f\left(\cdot ,u_{k_{n}}\left(\cdot \right),w_{k_{n}}\left(\cdot \right)\right)u_{k_{n}}\left(\cdot \right)-f\left(\cdot ,{\tilde{u}}_{0}\left(\cdot \right),w_{0}\left(\cdot \right)\right){\tilde{u}}_{0}\left(\cdot \right)\right|$$

$$\leq 2\left(\eta \left(\cdot \right)h_{1}\left(\cdot \right)+\alpha_{1}h_{1}^{r}\left(\cdot \right)+\alpha_{2}h_{1}\left(\cdot \right)h_{2}^{q/r^{\prime }}\left(\cdot \right)\right)\in L^{1}\left(\Omega \right).$$

Thus

(10) $$f\left(\cdot ,u_{k_{n}}\left(\cdot \right),w_{k_{n}}\left(\cdot \right)\right)u_{k_{n}}\left(\cdot \right)⟶f\left(\cdot ,{\tilde{u}}_{0}\left(\cdot \right),w_{0}\left(\cdot \right)\right){\tilde{u}}_{0}\left(\cdot \right)\;\;\mathrm{in}\;L^{1}\left(\Omega \right),$$

by Lebesgue’s dominated convergence theorem. A similar argument gives

(11) $$f\left(\cdot ,u_{k_{n}}\left(\cdot \right),w_{k_{n}}\left(\cdot \right)\right)v\left(\cdot \right)⟶f\left(\cdot ,{\tilde{u}}_{0}\left(\cdot \right),w_{0}\left(\cdot \right)\right)v\left(\cdot \right)\;\;\mathrm{in}\;L^{1}\left(\Omega \right)$$

for all $v\in X_{0}.$ Now, since $u_{k_{n}}$ is the weak solution to problem (8) with parameter $w_{k_{n}},$ we have

(12) $$\left(u_{k_{n}},v\right)-\int_{\Omega }f\left(x,u_{k_{n}},w_{k_{n}}\right)v\;dx=0$$

for all $v\in X_{0}$ and all n∈N. Hence

(13) $$\left({\tilde{u}}_{0},v\right)-\int_{\Omega }f\left(x,{\tilde{u}}_{0},w_{0}\right)v\;dx=0$$

for all $v\in X_{0},$ by (9) and (11). This means that ${\tilde{u}}_{0}$ is the weak solution to problem (8) corresponding to parameter $w_{0}.$ So ${\tilde{u}}_{0}=u_{0},$ by the uniqueness. Next, putting $v=u_{k_{n}}$ in (12), $v=u_{0}$ in (13), and using (10), we obtain

$$\begin{array}{ccc} {∥u_{k_{n}}∥}^{2}-{∥u_{0}∥}^{2} & = & \left(u_{k_{n}},u_{k_{n}}\right)-\left(u_{0},u_{0}\right)\\ & = & \int_{\Omega }f\left(x,u_{k_{n}},w_{k_{n}}\right)u_{k_{n}}\;dx-\int_{\Omega }f\left(x,u_{0},w_{0}\right)u_{0}\;dx⟶0 \end{array}$$

as n→∞. Additionally, since ${\left\{u_{k_{n}}\right\}}_{n\in N}$ weakly converges to $u_{0}$ and $X_{0}$ is the Hilbert space, we obtain $u_{k_{n}}\to u_{0}$ strongly in $X_{0}$. This proves the theorem. □

## 5. Conclusions

In this paper, we discussed the existence of the unique solution for the nonlocal equation, driven by the integrodifferential operator of fractional Laplacian type. We met the Hilbert and Courant requirements which demand not only the existence and uniqueness of the solution but also its continuous dependence on the date. We achieved this by imposing some monotonicity conditions on the source, which are optimal. Applying the obtained result to linear problem (7) led us to the necessary condition for the existence of a non-trivial solution, that is, the Lyapunov-type inequality, which estimates ${∥a∥}_{p}$ from below for $\frac{N}{2s}\leq p\leq \infty$. We also showed that by estimating the *p*-norm of *a* from below for $\frac{N}{2s}<p\leq \infty$ we cannot obtain sufficient conditions for the existence of a non-trivial solution. We left as an open problem (with some suggestions for answers) what can be said in the situation when 1≤p<(resp. $\leq )\frac{N}{2s}.$

## Data Availability

Not applicable.
